# Current Trends and Impact of Liver Biopsy on Survival in Hepatocellular Carcinoma: A Korean Multicenter Analysis

**DOI:** 10.3390/diagnostics15070818

**Published:** 2025-03-24

**Authors:** Seong Joon Chun, Jeong-Ju Yoo, Sang Gyune Kim, Young-Seok Kim

**Affiliations:** 1 Division of Gastroenterology and Hepatology, Department of Internal Medicine, Inha University, Bucheon 14584, Republic of Korea; coordinac21@naver.com; 2 Bucheon Hospital, Korean Liver Cancer Association, Bucheon 14584, Republic of Korea; puby17@naver.com (J.-J.Y.); mcnulty@schmc.ac.kr (S.G.K.); 3 Division of Gastroenterology and Hepatology, Department of Internal Medicine, Soonchunhyang University Hospital, Bucheon 14584, Republic of Korea

**Keywords:** hepatocellular carcinoma, biopsy, survival outcomes

## Abstract

**Background/Objectives**: The diagnosis of hepatocellular carcinoma (HCC) mainly relies on imaging, with biopsy reserved for cases where imaging results are inconclusive. While biopsy offers histological confirmation and can guide treatment decisions, its impact on survival outcomes in HCC patients remains uncertain. This study aimed to examine biopsy practices and evaluate their effects on survival rates in HCC patients. **Methods**: We analyzed data from 18,304 HCC patients in the Korean Primary Liver Cancer Registry from 2008 to 2019. We compared overall survival (OS) and transplant-free survival (TFS) between patients who underwent a biopsy and those diagnosed solely based on imaging. **Results**: From 2008 to 2019, liver biopsy rates varied, reaching a peak of 12.44% in 2009 and declining to 8.18% in 2012, with the majority of patients (90.3%) diagnosed through imaging. Trans-arterial chemoembolization was the most common treatment overall (40.5%), especially in the non-biopsy group. Sorafenib use increased significantly in both groups after 2015. Patients who underwent biopsy had lower OS (43.1 ± 1.29 months) and TFS (42.45 ± 1.28 months) compared to those diagnosed via imaging (OS: 54.5 ± 0.48 months, TFS: 52.57 ± 0.47 months, *p* < 0.001 for both). However, Cox regression analysis indicated that biopsy was not a significant risk factor for OS (HR: 1.021, *p* = 0.502) or TFS (HR: 1.013, *p* = 0.674). Subgroup analysis suggested that biopsy may benefit patients with advanced stage IV-B by enabling more aggressive treatment strategies. **Conclusions**: Liver biopsy rates fluctuated over time, with the majority of HCC diagnoses made through imaging. Although biopsy does not significantly affect OS or TFS, it may provide advantages in advanced cases, such as stage IV-B, by guiding more aggressive treatment strategies.

## 1. Introduction

Hepatocellular carcinoma (HCC) has emerged as a significant global public health concern, ranking as the sixth most common cancer worldwide and the third leading cause of cancer-related deaths [1,2]. Unlike most other solid tumors, HCC is not routinely diagnosed through biopsy [3]. Instead, diagnosis is often based on imaging features, such as arterial phase hyper-enhancement and delayed washout observed on computed tomography (CT) and magnetic resonance imaging (MRI) scans [4]. These findings demonstrate high sensitivity and specificity in cirrhotic patients, allowing for diagnosis based on imaging alone in many cases, without the need for biopsy [5,6]. Guidelines from the American Association for the Study of Liver Diseases (AASLD) and the European Association for the Study of the Liver (EASL) also recommend imaging as the primary diagnostic tool for HCC, reserving biopsy for cases where imaging findings are inconclusive [7,8].

However, imaging alone does not always provide an accurate diagnosis for all HCC cases. Patients without cirrhosis, or those with HCC arising from fatty liver, are less likely to display typical imaging features, complicating diagnosis [1,9]. In non-cirrhosis patients, the accuracy of imaging for liver cancer diagnosis decreases, often making biopsy necessary [8]. Additionally, small tumors less than 1 cm in size are often challenging to diagnose with imaging alone, making biopsy an essential diagnostic tool in such cases [7,10]. Furthermore, imaging may not always differentiate between HCC and other liver cancers, such as intrahepatic cholangiocarcinoma (iCCA), where biopsy can provide additional diagnostic insight [11,12]. Biopsy also plays a critical role in diagnosing mixed liver cancers and other hepatic tumors [7,13].

Recent advances in targeted therapies and immunotherapies have made precision medicine approaches increasingly important in HCC treatment [14,15]. These therapies enable personalized treatment based on the molecular characteristics of the tumor [7,16]. Biopsy-obtained tumor tissue enables genetic analysis, offering critical information for developing targeted therapies and shaping treatment strategies for HCC [17]. Consequently, biopsy is expected to play an increasingly important role in future HCC treatment [11]. However, due to concerns that biopsy-related complications could worsen HCC prognosis, its use remains limited in clinical practice [8].

In this study, we analyzed multicenter data from Korea between 2008 and 2019 to evaluate the frequency of biopsies and their impact on survival rates in HCC patients. The primary goal of the study is to assess the effect of biopsy on overall survival (OS) and transplant-free survival (TFS) in HCC patients. Additionally, we examined the clinical implications in which biopsy may become more necessary.

## 2. Materials and Methods

### 2.1. Data Collection

The study analyzed data from 18,304 patients diagnosed with liver cancer and registered in the Korean Primary Liver Cancer Registry (KPLCR) between January 2008 and December 2019. The KPLCR is a carefully curated subset of the broader Korean Central Cancer Registry (KCCR), which captures over 95% of all cancer cases in South Korea [18,19]. The KPLCR employs a sophisticated two-stage sampling method to select approximately 15% of newly diagnosed primary liver cancer patients from the KCCR, ensuring an accurate reflection of the HCC distribution nationwide. Through this process, approximately 1500 patients are randomly registered in the KPLCR each year. Patients were diagnosed with liver cancer through either biopsy or imaging techniques such as CT or MRI. The collected data included clinical characteristics such as age, sex, date of diagnosis, blood test results, tumor characteristics (size, number, invasiveness, metastasis, etc.), and whether a biopsy was performed. Additionally, the treatment methods administrated to the patients were recorded [18,20].

### 2.2. Group Classification

To assess the frequency of biopsies performed in patients diagnosed with HCC through imaging in Korea, we first examined the biopsy rates during the study period. We analyzed annual trends in these rates, as well as biopsy rates categorized by treatment methods.

Next, we classified the patients into two groups: those who underwent biopsy at the time of HCC diagnosis and those who did not. We then compared the baseline characteristics of the biopsy group and the non-biopsy group using the collected data. The factors analyzed included age, sex, height, weight, laboratory findings (including platelet count, AST, ALT, total bilirubin, creatinine, albumin, prothrombin time—international normalized ratio (PT-INR)), and modified Union for International Cancer Control (mUICC) stage to identify any differences between the two groups. Additionally, we examined whether there were differences in the treatment methods based on biopsy status. Given the increase in sorafenib prescriptions following its introduction in Korea in 2007, we also compared treatment methods before and after 2015. To assess the impact of biopsy on the prognosis of HCC, we analyzed and compared the OS between the two groups. We also compared liver TFS to account for the differences influenced by liver transplantation. Subgroup analyses were conducted based on the mUICC stage, Barcelona Clinic Liver Cancer (BCLC) stage, treatment methods, and the years before or after 2015, when sorafenib became widely used in Korea.

### 2.3. Statistical Analysis

The data are presented as numbers (percentages) or mean ± standard deviation, as appropriate. The significance of differences among continuous and categorical variables was examined using the Student *t*-test (or Mann–Whitney U test) and chi-squared test (or Fisher’s exact test), respectively. OS of patients was evaluated using the Kaplan–Meier method, with the survival differences compared using the log-rank test. A Cox regression analysis was performed to assess the association between OS and the variables, calculating hazard ratios (HRs) and their 95% confidence intervals (CIs). All statistical analyses were conducted using SPSS ver. 26.0 (IBM Corp., Armonk, NY, USA). Two-sided *p* values < 0.05 were considered to indicate statistical significance.

## 3. Results

### 3.1. Patient Characteristics

A total of 18,304 patients diagnosed with HCC were registered in the KPLCR between January 2008 and December 2019. Among them, 1776 patients (9.7%) had undergone a liver biopsy prior to diagnosis, while 16,528 patients (90.3%) were diagnosed through imaging tests without a biopsy. The baseline characteristics of the patients are summarized in Table 1. The biopsy group had a higher average age (61.28 ± 11.56 vs. 62.14 ± 12.12 years, *p* = 0.004) and a greater proportion of males (81.96% vs. 78.91%, *p* = 0.003).

According to blood test results, the biopsy group had significantly higher platelet counts (192.11 ± 95.35 vs. 159.04 ± 89.33, *p* < 0.001) and lower levels of total bilirubin (1.45 ± 2.83 vs. 1.68 ± 3.17, *p* = 0.003) and PT-INR (1.11 ± 0.16 vs. 1.17 ± 0.23, *p* < 0.0001) compared to the non-biopsy group. No significant differences were observed in other laboratory results, such as AST or ALT. Additionally, a higher proportion of patients in the biopsy group were in the advanced stages of the disease, particularly in stage IV-B (19.57% vs. 9.01%, *p* < 0.001).

### 3.2. Changes in Biopsy Rates over Time

**Figure 1:**
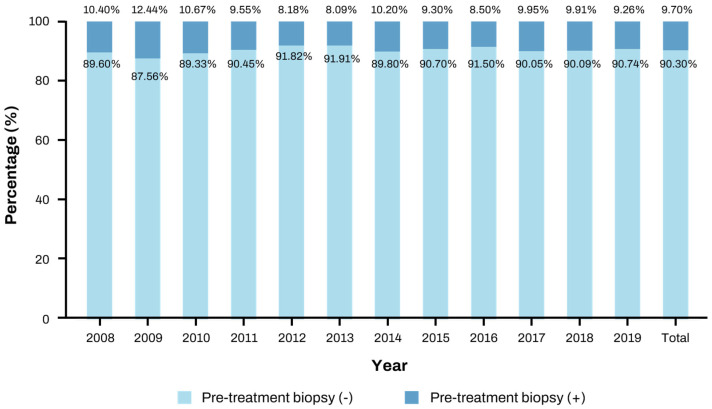
Percentages of pre-treatment biopsies by year.

The proportion of patients who underwent liver biopsy before treatment fluctuated slightly from 2008 to 2019. The highest biopsy rate was recorded in 2009, at 12.44%, while the lowest was observed in 2012, at 8.18%. Overall, the majority of patients (90.30%) were diagnosed through imaging tests, with only 9.70% undergoing biopsy (Table 2). These yearly trends are shown in Figure 1, which shows no consistent increase or decrease over time. 

### 3.3. First-Line Treatment Choices for HCC Based on Biopsy Status

From 2008 to 2019, trans-arterial chemoembolization (TACE) was the most frequently used treatment for HCC, applied in 40.5% of cases. This was followed by resection (20.4%), best supportive care (19.6%), local ablation (10.7%), systemic chemotherapy including sorafenib (6.3%), radiotherapy (1.5%), and liver transplantation (LT, 1.0%) as the first-line treatment.

**Figure 2:**
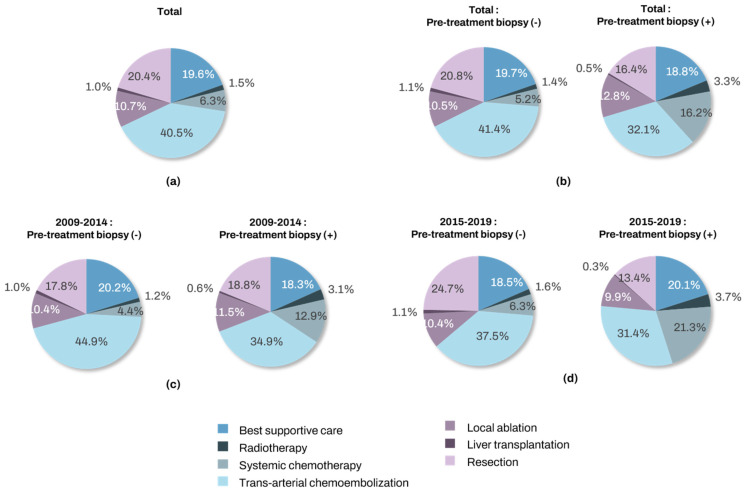
Pie chart illustrating treatment distribution including totals and by time period. (**a**) Total, (**b**) pre-treatment biopsy (+) vs. pre-treatment biopsy (−) groups, (**c**) before the sorafenib period, and (**d**) after the sorafenib period.

When comparing treatment methods by biopsy status, TACE was more frequently used in the non-biopsy group (41.4% vs. 32.8%), and resection was also more common in this group (20.8% vs. 16.2%). Conversely, sorafenib was used more often in the biopsy group (16.0% vs. 5.2%). There were changes in treatment patterns between 2008–2014 and 2015–2019. The use of systemic chemotherapy, including sorafenib, increased in both groups to 21.3%. Although TACE remained the most commonly used treatment, its utilization slightly decreased compared to the previous period (Figure 2).

### 3.4. Comparison of OS and TFS Based on Biopsy Status

**Figure 3:**
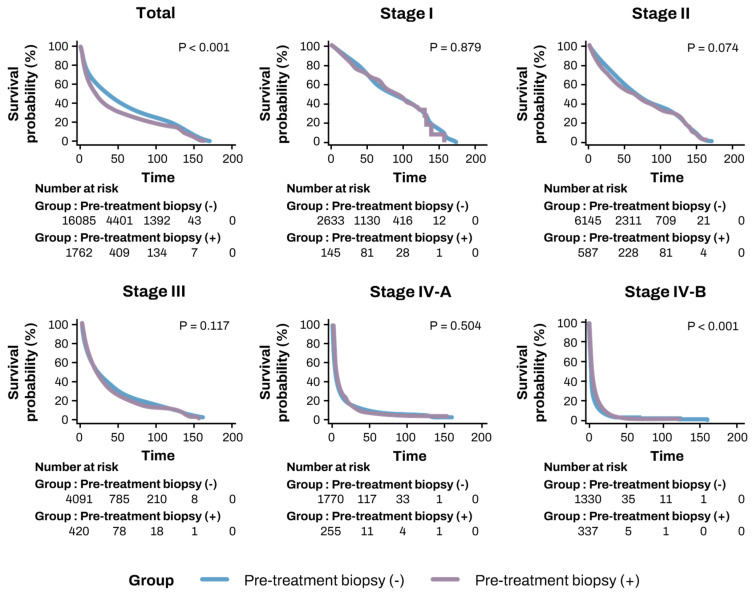
Kaplan–Meier survival curves comparing pre-treatment biopsy (+) vs. pre-treatment biopsy (−) groups for overall survival.

Kaplan–Meier analysis revealed that the biopsy group had significantly lower OS compared to the non-biopsy group (43.1 ± 1.29 months vs. 54.5 ± 0.48 months, *p* < 0.001). Similarly, TFS was significantly lower in the biopsy group compared to the non-biopsy group (42.45 ± 1.28 months vs. 52.57 ± 0.47 months, *p* < 0.001) (Table 2, Figure 3). Subgroup analysis was performed according to mUICC and BCLC stages (Table 2 and Table 3). In the subgroup analysis by mUICC stage, the patients in stage IV-B exhibited higher OS rates in the biopsy group (9.08 ± 0.84 vs. 8.21 ± 0.60 months, *p* < 0.001). However, no significant differences were observed between the two groups in other stages or in the BCLC classification.

Next, we classified patients based on their first-line treatment and compared OS and TFS according to biopsy status. In the TACE and radiotherapy groups, OS and TFS were higher in the non-biopsy group compared to the biopsy group. However, in the other treatment groups, biopsy status did not significantly affect prognosis (Table 2 and Table 3).

### 3.5. Risk Factors for OS and TFS

To evaluate independent risk factors for OS and TFS, multivariate Cox regression analysis was performed (Table 4 and Table 5). After adjusting for other variables such as age, sex, mUICC stage, Child–Pugh score, and treatment method, pre-treatment liver biopsy was not identified as an independent risk factor for either OS (HR: 1.021, 95% CI: 0.961–1.085, *p* = 0.504) or TFS (HR: 1.013, 95% CI: 0.954–1.076, *p* = 0.674). Conversely, advanced mUICC stage and higher Child–Pugh scores were confirmed as the most significant prognostic factors (*p* < 0.001) (Table 4 and Table 5).

## 4. Discussion

This study is a multicenter analysis designed to evaluate the current use of liver biopsy in the diagnosis of HCC and its impact on survival outcomes, specifically focusing on OS and TFS.

The first major finding of our study is related to the real-world application of liver biopsy in HCC diagnosis. We observed that the majority of patients (90.30%) were diagnosed using imaging techniques, with only 9.70% undergoing a biopsy. This suggests a strong preference in Korean clinical practice for imaging-based diagnosis, which is likely guided by local guidelines, particularly for patients with liver cirrhosis. Notably, biopsy rates remained consistently low over the years, even after the introduction of targeted therapies such as sorafenib. In contrast, a study from the U.S. and Puerto Rico reported a biopsy rate of 29.6%, indicating greater reliance on biopsy in those regions, potentially due to different diagnostic practices or regulatory environment [21]. Additionally, we observed differences in first-line treatment choices based on biopsy status. TACE was more frequently used in the non-biopsy group (41.4% vs. 32.8%), while sorafenib and radiotherapy were more commonly applied in the biopsy group. This finding suggests that patients undergoing biopsy were more likely to receive aggressive or advanced treatments, possibly reflecting their more advanced disease stages at the time of diagnosis.

Our second finding indicates that biopsy status did not significantly affect OS or TFS. Initially, patients who did not undergo a biopsy demonstrated better OS and TFS than those who did. However, this outcome seems to be confounded by disease stage, as a significantly higher proportion of patients in the biopsy group were diagnosed with advanced-stage disease, particularly stage IV-B (19.57% vs. 9.01%, *p* < 0.001). After adjusting for disease stage and other confounding variables, biopsy was not found to be an independent risk factor for either OS (HR: 1.021, *p* = 0.502) or TFS (HR: 1.013, *p* = 0.674). Our results align with previous studies, some of which have shown no impact of biopsy on survival, while others have suggested worse outcomes for biopsy patients [5,21]. Possible reasons for the lack of survival benefit from biopsy in our study include (1) the advanced-stage bias in the biopsy group, (2) the potential risks associated with biopsy, such as complications or delays in treatment [22], (3) the high diagnostic accuracy of imaging techniques in early-stage disease, and (4) the possibility that many biopsy patients did not receive targeted therapies that could have leveraged molecular information derived from biopsy results.

The third important finding of our study is that despite the overall lack of a survival benefit from biopsy, it appears to be useful in patients with stage IV-B HCC. Our subgroup analysis indicated that in these advanced cases, biopsy could aid in guiding more aggressive treatment strategies, including systemic chemotherapy with sorafenib or radiotherapy, following histological confirmation of HCC. These findings underscore the clinical utility of biopsy in situations where imaging alone may be insufficient for making critical treatment decisions. While molecular markers were not extensively used to guide treatment during the sorafenib era [23,24], the emergence of immune checkpoint inhibitors like atezolizumab has transformed the treatment landscape [25]. In the immunotherapy era, biopsy could assume a more significant role, as it facilitates molecular profiling and personalized treatment based on the tumor’s specific characteristics [17]. This enhanced capability makes biopsy potentially more valuable in determining prognosis and optimizing treatment for advanced-stage HCC patients.

Despite these potential advantages, the current low rate of biopsy implementation persists due to concerns about complications such as bleeding and tumor seeding. However, advancements in technology have significantly improved the safety of biopsies. Recent studies indicate that the risks of bleeding and tumor seeding during biopsy have dramatically decreased, thanks to improved needle technology and increased procedural expertise. As a result, biopsy can now be performed relatively safely, even in high-risk patients [22,26].

Our study has several limitations. First, since this is an observational study, the results are focused on explaining the association, not the causal relationship. The lower overall survival rate (OS) and probability of survival without transplantation (TFS) in the biopsy-treated patient population are also likely due to selection bias. However, multivariate analysis, after correcting for confounding variables, showed that biopsy was not identified as an independent risk factor for OS or TFS. Nevertheless, the effects of residual confounding cannot be completely excluded, and a prospective study is needed in the future to determine the effect of biopsy on survival. Second, an imbalance in the sample size between the biopsy and non-biopsy groups may introduce statistical bias, particularly in subgroup analyses. Third, we were unable to analyze data from the era of immune checkpoint inhibitors, which limits our conclusions regarding the role of biopsy in modern immunotherapy treatments. Finally, biopsy-related complications, such as bleeding or tumor seeding, were not included in the analysis, as our dataset did not contain specific information on these adverse events. According to previous studies, the risk of bleeding after biopsy was reported to be 0.2%–2.7%, the incidence of tumor seeding was approximately 0.5%, and the incidence of biopsy-associated mortality was less than 0.1% [22,26]. Although these risks are relatively low, concerns about biopsy-related complications may be one of the factors for the limited use of biopsy in diagnosing liver cancer. Future studies need to include specific incidences of biopsy complications for a more comprehensive risk–benefit analysis. Despite these limitations, our study has the strength of analyzing a large dataset of 18,304 patients from multiple centers. It reflects real-world clinical practice and provides insight into the national landscape of HCC management in the hepatitis B virus endemic area. Future studies should focus on the evolving role of liver biopsy in the context of immunotherapy and targeted therapies. Prospective studies that incorporate molecular markers and immune-based treatments will be essential in determining the role of biopsy in personalized treatment strategies for HCC.

This study confirms that liver biopsy does not significantly affect prognosis in the majority of HCC cases. However, it may provide a survival benefit for advanced-stage disease patients. As molecular analysis gains increasing importance in the era of immunotherapy, the role of biopsy could expand, positioning it as a valuable tool in the realm of precision medicine for HCC.

## Figures and Tables

**Table 1:** Baseline characteristics.

Variable	All (n = 18,304)	Pre-Treatment Biopsy (−) of Liver Cancer (n = 16,528)	Pre-Treatment Biopsy (+) of Liver Cancer (n = 1776)	*p*
Age (years)	61.36 ± 11.61	61.28 ± 11.56	62.14 ± 12.12	0.004
Male	14,497 (79.20%)	13,042 (78.91%)	1455 (81.96%)	0.003
Height (cm)	164.47 ± 8.36	164.48 ± 8.38	164.39 ± 8.19	0.680
Weight (kg)	64.97 ± 11.25	65.10 ± 11.25	63.84 ± 11.19	<0.001
Laboratory findings				
Platelet (10^9^/L)	162.28 ± 90.47	159.04 ± 89.33	192.11 ± 95.35	<0.001
AST (IU/L)	79.16 ± 122.42	74.94 ± 89.37	76.36 ± 4.35	0.877
ALT (IU/L)	56.13 ± 109.27	55.79 ± 96.01	59.27 ± 192.54	0.207
Total bilirubin (mg/dL)	1.66 ± 3.05	1.68 ± 3.07	1.45 ± 2.83	0.003
Creatinine (mg/dL)	0.98 ± 0.78	0.99 ± 0.80	0.95 ± 0.55	0.066
Albumin (mg/dL)	3.74 ± 0.67	3.74 ± 0.68	3.81 ± 0.60	<0.001
PT-INR	1.16 ± 0.60	1.17 ± 0.23	1.11 ± 0.16	<0.001
mUICC stage				
Stage I	2787 (15.34%)	2642 (16.10%)	145 (8.25%)	<0.001
Stage II	6787 (37.37%)	6198 (37.78%)	589 (33.50%)	<0.001
Stage III	4626 (25.47%)	4204 (25.62%)	422 (24.00%)	<0.001
Stage IV-A	2142 (11.79%)	1884 (11.48%)	258 (14.68%)	<0.001
Stage IV-B	1822 (10.03%)	1478 (9.01%)	344 (19.57%)	<0.001
BCLC stage				
Stage 0	1400 (9.07%)	1310 (9.45%)	90 (8.25%)	<0.001
Stage A	3928 (25.46%)	3669 (26.47%)	259 (16.49%)	<0.001
Stage B	2882 (18.68%)	4204 (18.47%)	322 (20.50%)	<0.001
Stage C	6036 (39.12%)	1884 (37.48%)	841 (53.53%)	<0.001
Stage D	1184 (7.67%)	1478 (8.12%)	59 (3.76%)	<0.001

Data are presented as mean ± standard deviation for continuous variables, or as number (percentage) for categorical variables. Abbreviations: ALT, alanine aminotransferase; AST, aspartate aminotransferase; PT-INR, prothrombin time with international normalized ratio.

**Table 2:** Comparison of overall survival rates in patients with and without pre-treatment liver biopsy.

Group	Total (n = 18,304)	Pre-Treatment Biopsy (−) of Liver Cancer (n = 16,528)	Pre-Treatment Biopsy (+) of Liver Cancer (n = 1776)	*p*
Total	53.47 ± 0.45	54.58 ± 0.48	43.13 ± 1.29	<0.001
mUICC stage				
Stage I (n = 2787)	88.49 ± 1.35	88.66 ± 1.40	86.40 ± 4.80	0.879
Stage II (n = 6787)	73.92 ± 0.77	74.22 ± 0.81	70.24 ± 2.52	0.074
Stage III (n = 4626)	39.26 ± 0.71	39.60 ± 0.75	35.93 ± 2.20	0.117
Stage IV-A (n = 2142)	15.12 ± 0.68	15.11 ± 0.72	14.63 ± 1.84	0.504
Stage IV-B (n = 1822)	8.42 ± 0.53	8.21 ± 0.60	9.08 ± 0.84	<0.001
BCLC stage				
Stage 0 (n = 1400)	100.29 ± 1.82	101.02 ± 1.91	91.67 ± 6.08	0.247
Stage A (n = 3928)	84.67 ± 1.03	84.25 ± 1.08	88.47 ± 3.65	0.233
Stage B (n = 2882)	47.36 ± 0.97	48.38 ± 1.04	38.30 ± 2.35	0.001
Stage C (n = 6036)	28.04 ± 0.58	28.68 ± 0.63	23.75 ± 1.37	0.004
Stage D (n = 1184)	14.13 ± 0.49	14.20 ± 1.06	10.70 ± 2.21	0.300
1st line treatment				
Resection (n = 3726)	98.98 ± 1.17	99.11 ± 1.23	95.78 ± 3.85	0.383
Liver transplantation (n = 182)	107.33 ± 5.18	107.27 ± 5.33	87.375 ± 20.01	0.801
Radiofrequency ablation (n = 1963)	86.06 ± 1.41	86.64 ± 1.54	80.87 ± 3.83	0.112
Trans-arterial chemoembolization (n = 7424)	48.92 ± 0.59	50.06 ± 0.62	36.11 ± 1.72	<0.001
Systemic chemotherapy (n = 1146)	12.66 ± 0.76	12.49 ± 0.87	13.08 ± 1.52	0.423
Radiotherapy (n = 283)	22.71 ± 2.57	24.80 ± 2.97	10.37 ± 1.45	0.035
Best supportive care (n = 3580)	14.23 ± 0.52	14.23 ± 0.54	14.03 ± 1.50	0.112
Years of diagnosis				
2008–2014 (n = 10,733)	55.10 ± 0.56	55.72 ± 0.59	49.43 ± 1.71	0.001
2015–2018 (n = 7571)	34.06 ± 0.35	35.13 ± 0.37	23.89 ± 0.96	<0.001

Data are presented as mean ± standard deviation. All survival rates are reported in months. Abbreviations: mUICC, Modified Union for International Cancer Control; BCLC, Barcelona Clinic Liver Cancer.

**Table 3:** Comparison of transplant-free survival rates in patients with and without pre-treatment liver biopsy (Kaplan–Meier analysis).

Group	Total (n = 18,304)	Pre-Treatment Biopsy (−) of Liver Cancer (n = 16,528)	Pre-Treatment Biopsy (+) of Liver Cancer (n = 1776)	*p*
Total	51.59 ± 0.44	52.57 ± 0.47	42.45 ± 1.28	<0.001
mUICC stage				
Stage I (n = 2787)	85.66 ± 1.34	85.81 ± 1.40	83.9 ± 4.81	0.902
Stage II (n = 6787)	71.55 ± 0.76	71.62 ± 0.80	70.24 ± 2.52	0.486
Stage III (n = 4626)	37.53 ± 0.69	37.82 ± 0.73	34.77 ± 2.16	0.202
Stage IV-A (n = 2142)	14.77 ± 0.66	14.89 ± 0.72	13.32 ± 1.62	0.733
Stage IV-B (n = 1822)	8.41 ± 0.53	8.20 ± 0.60	9.20 ± 0.84	<0.001
BCLC stage				
Stage 0 (n = 1400)	98.88 ± 1.82	99.68 ± 1.91	89.27 ± 6.09	0.171
Stage A (n = 3928)	82.33 ± 1.03	81.79 ± 1.08	87.69 ± 3.67	0.105
Stage B (n = 2882)	45.88 ± 0.97	46.78 ± 1.02	37.75 ± 2.33	0.003
Stage C (n = 6036)	27.11 ± 0.58	27.71 ± 0.62	23.11 ± 1.34	0.008
Stage D (n = 1184)	10.19 ± 0.49	10.14 ± 0.80	10.74 ± 2.21	0.045
1st line treatment				
Resection (n = 3726)	98.49 ± 1.17	98.59 ± 1.23	95.79 ± 3.85	0.470
Radiofrequency ablation (n = 1963)	84.89 ± 1.40	85.37 ± 1.53	80.44 ± 3.82	0.168
Trans-arterial chemoembolization (n = 7424)	47.51 ± 0.58	48.59 ± 1.04	36.00 ± 1.72	<0.001
Systemic chemotherapy (n = 1146)	11.68 ± 0.68	15.15 ± 2.50	14.19 ± 1.50	0.911
Radiotherapy (n = 283)	21.56 ± 2.47	23.42 ± 1.29	10.37 ± 1.46	0.051
Best supportive care (n = 3580)	14.23 ± 0.52	14.23 ± 0.55	14.18 ± 1.51	0.112
Years of diagnosis				
2008–2014 (n = 10,733)	53.26 ± 0.55	53.78 ± 0.58	48.50 ± 1.69	0.015
2015–2018 (n = 7571)	33.10 ± 0.35	34.08 ± 0.37	23.77 ± 0.96	<0.001

Data are presented as mean ± standard deviation. All survival rates are reported in months. Abbreviations: mUICC, Modified Union for International Cancer Control; BCLC, Barcelona Clinic Liver Cancer.

**Table 4:** Cox regression analysis for overall survival.

	Univariate		Multivariate	
	HR (95% CI)	*p*	HR (95% CI)	*p*
Biopsy				
Pre-treatment biopsy (−)	1 (ref)		1 (ref)	
Pre-treatment biopsy (+)	1.287 (1.217–1.361)	<0.001	1.021 (0.961–1.085)	0.502
Age	1.017 (1.016–1.019)	<0.001	1.030 (1.013–1.011)	<0.001
Sex				
Female	1 (ref)		1 (ref)	
Male	1.160 (1.109–1.212)	<0.001	1.091 (1.040–1.145)	<0.001
Etiology				
HBV	1 (ref)		1 (ref)	
HCV	1.263 (1.193–1.338)	<0.001	1.072 (1.003–1.146)	0.040
Non-B, Non-C	1.334 (1.281–1.389)	<0.001	1.050 (1.003–1.098)	0.036
mUICC stage				
Stage I	1 (ref)		1 (ref)	
Stage II	1.401 (1.313–1.495)	<0.001	1.413 (1.316–1.517)	<0.001
Stage III	3.131 (2.933–3.342)	<0.001	2.407 (2.238–2.589)	<0.001
StageIV-A	7.442 (6.921–8.002)	<0.001	4.519 (4.162–4.907)	<0.001
StageIV-B	11.423 (10.589–12.322)	<0.001	5.970 (5.471–6.514)	<0.001
ChildPugh class				
Class A	1 (ref)		1 (ref)	
Class B	2.763 (2.652–2.879)	<0.001	1.820 (1.741–1.902)	<0.001
Class C	4.650 (4.318–5.007)	<0.001	3.021 (2.786–3.276)	<0.001
1st line Treatment				
Best supportive care	1 (ref)		1 (ref)	
Resection	0.097 (0.091−0.104)	<0.001	0.194 (0.180–0.209)	<0.001
Liver transplantation	0.094 (0.072–0.122)	<0.001	0.114 (0.087–0.150)	<0.001
Radiofrequency ablation	0.132 (0.123–0.143)	<0.001	0.284 (0.261–0.310)	<0.001
Trans-arterial chemoembolization	0.305 (0.292–0.319)	<0.001	0.444 (0.422–0.467)	<0.001
Systemic chemotherapy	1.028 (0.960–1.101)	0.429	0.823 (0.763–0.887)	<0.001
Radiotherapy	0.702 (0.617–0.800)	<0.001	0.739 (0.644–0.846)	<0.001

Abbreviations: HR, hazard ratio; CI, confidence interval; HBV, hepatitis B virus; HCV, hepatitis C virus.

**Table 5:** Cox regression analysis for transplant-free survival.

	Univariate		Multivariate	
	HR (95% CI)	*p*	HR (95% CI)	*p*
Biopsy				
Pre-treatment biopsy (−)	1 (ref)		1 (ref)	
Pre-treatment biopsy (+)	1.247 (1.180–1.319)	<0.001	1.013 (0.954–1.076)	0.674
Age	1.015 (1.013–1.016)	<0.001	1.012 (1.010–1.013)	<0.001
Sex				
Female	1 (ref)		1 (ref)	
Male	1.075 (1.052–1.099)	<0.001	1.081 (1.030–1.133)	<0.001
Etiology				
HBV	1 (ref)		1 (ref)	
HCV	1.228 (1.160–1.301)	<0.001	1.061 (0.997–1.129)	0.060
Non-B, Non-C	1.293 (1.243–1.346)	<0.001	1.036 (0.991–1.084)	0.122
mUICC stage				
Stage I	1 (ref)		1 (ref)	
Stage II	1.376 (1.291–1.467)	<0.001	1.415 (1.320–1.517)	<0.001
Stage III	3.031 (2.843–3.232)	<0.001	2.352 (2.190–2.525)	<0.001
StageIV-A	6.891 (6.415–7.402)	<0.001	4.289 (3.955–4.651)	<0.001
StageIV-B	10.317 (9.574–11.117)	<0.001	5.660 (5.193–6.169)	<0.001
ChildPugh class				
Class A	1 (ref)		1 (ref)	
Class B	2.898 (2.783–3.019)	<0.001	1.855 (1.775–1.938)	<0.001
Class C	5.696 (5.294–6.129)	<0.001	2.994 (2.763–3.244)	<0.001
1st-line Treatment				
Best supportive care	1 (ref)		1 (ref)	
Resection	0.099 (0.093–0.106)	<0.001	0.193 (0.179–0.208)	<0.001
Liver transplantation	4.308 (3.701–5.014)	<0.001	7.213 (6.133–8.412)	<0.001
Radiofrequency ablation	0.137 (0.127–0.147)	<0.001	0.287 (0.263–0.312)	<0.001
Trans-arterial chemoembolization	0.316 (0.302–0.330)	<0.001	0.454 (0.432–0.478)	<0.001
Systemic chemotherapy	1.064 (0.993–1.139)	0.077	0.849 (0.787–0.915)	<0.001
Radiotherapy	0.724 (0.636–0.824)	<0.001	0.757 (0.661–0.868)	<0.001

Abbreviations: HR, hazard ratio; CI, confidence interval; HBV, hepatitis B virus; HCV, hepatitis C virus.

## Data Availability

The datasets generated and/or analyzed during the current study are available from the corresponding author upon reasonable request.

## Abbreviations

The following abbreviations are used in this manuscript:

AASLD	American Association for the Study of Liver Diseases
BCLC	Barcelona Clinic Liver Cancer
CI	Confidence Interval
CT	Computed Tomography
HCC	Hepatocellular Carcinoma
HR	Hazard Ratio
iCCA	Intrahepatic Cholangiocarcinoma
IQR	Interquartile Range
KPLCR	Korean Primary Liver Cancer Registry
LT	Liver Transplantation
MRI	Magnetic Resonance Imaging
mUICC	Modified Union for International Cancer Control
OS	Overall Survival
PT-INR	Prothrombin Time-International Normalized Ratio
SPSS	Statistical Package for the Social Sciences
TACE	Trans-Arterial Chemoembolization
TFS	Transplant-Free Survival
mUICC	Modified Union for International Cancer Control
